# Penta­potassium praseodymium(III) dilithium deca­fluoride, K_5_PrLi_2_F_10_

**DOI:** 10.1107/S1600536809044043

**Published:** 2009-10-28

**Authors:** Anna Gagor

**Affiliations:** a W. Trzebiatowski Institute of Low Temperature and Structure Research, Polish Academy of Sciences, Okólna str. 2, PO Box 1410, 50-950 Wrocław, Poland

## Abstract

The crystal structure of K_5_PrLi_2_F_10_ is isotypic with those of other K_5_ *RE*Li_2_F_10_ compounds (*RE* = Eu, Nd). The lanthanoid ions are isolated in K_5_PrLi_2_F_10_, with a mean separation between the Pr ions of 7.356 Å. It classifies this crystal as a so-called self-activated material containing lanthanoid ions within the matrix. Except for two K^+^ and two F^−^ ions, all atoms are located on sites with *m* symmetry. In the structure, distorted PrF_8_ dodeca­hedra and two different LiF_4_ tetra­hedra share F atoms, forming sheets parallel to (100). The isolated PrF_8_ dodeca­hedra exhibit a mean Pr—F distance of 2.406 Å. The K^+^ cations are located within and between these sheets, leading to highly irregular KF_*x*_ polyhedra with coordination numbers of eight and nine for the alkali metal cations.

## Related literature

The structures of the isotypic Nd and Eu analogues have been reported by Hong & McCollum (1979 ▶) and Gagor (2009 ▶). For background to bond-valence calculations, see: Brown (1992 ▶, 2002 ▶); Mattausch *et al.* (1991 ▶). Synthetic details were described by Ryba-Romanowski *et al.* (2007 ▶).

## Experimental

### 

#### Crystal data

K_5_PrLi_2_F_10_
*M* *_r_* = 540.29
Orthorhombic, 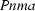

*a* = 20.6492 (6) Å
*b* = 7.7903 (3) Å
*c* = 6.9255 (2) Å
*V* = 1114.06 (6) Å^3^
*Z* = 4
Mo *K*α radiation
μ = 6.34 mm^−1^
*T* = 295 K
0.35 × 0.13 × 0.05 mm

#### Data collection

Kuma KM-4 with CCD area-detector diffractometer
Absorption correction: multi-scan (*CrysAlis RED*; Oxford Diffraction, 2007 ▶) *T*_min_ = 0.40, *T*_max_ = 0.73
24749 measured reflections
4910 independent reflections
3709 reflections with *I* > 2σ(*I*)
*R*_int_ = 0.045

#### Refinement

*R*[*F* ^2^ > 2σ(*F* ^2^)] = 0.029
*wR*(*F* ^2^) = 0.045
*S* = 1.02
4910 reflections
98 parameters
Δρ_max_ = 1.17 e Å^−3^
Δρ_min_ = −2.04 e Å^−3^

### 

Data collection: *CrysAlis CCD* (Oxford Diffraction, 2007 ▶); cell refinement: *CrysAlis RED* (Oxford Diffraction, 2007 ▶); data reduction: *CrysAlis RED*; program(s) used to solve structure: *SHELXS97* (Sheldrick, 2008 ▶); program(s) used to refine structure: *SHELXL97* (Sheldrick, 2008 ▶); molecular graphics: *DIAMOND* (Brandenburg & Putz, 2006 ▶); software used to prepare material for publication: *SHELXL97*.

## Supplementary Material

Crystal structure: contains datablocks I, global. DOI: 10.1107/S1600536809044043/wm2268sup1.cif

Structure factors: contains datablocks I. DOI: 10.1107/S1600536809044043/wm2268Isup2.hkl

Additional supplementary materials:  crystallographic information; 3D view; checkCIF report

## Figures and Tables

**Table 1:** Selected bond lengths (Å)

Pr1—F5	2.3426 (12)
Pr1—F3^i^	2.3737 (8)
Pr1—F2	2.3751 (12)
Pr1—F1	2.4368 (11)
Pr1—F8^ii^	2.4463 (11)
Pr1—F4	2.4488 (8)
Li1—F6^iii^	1.802 (4)
Li1—F1^iv^	1.856 (4)
Li1—F3^v^	1.8602 (19)
Li2—F7	1.796 (4)
Li2—F4^iii^	1.819 (2)
Li2—F8	1.843 (4)

Symmetry codes: (i) 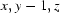; (ii) 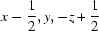; (iii) 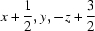; (iv) 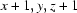; (v) 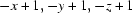.

**Fig. 1.**
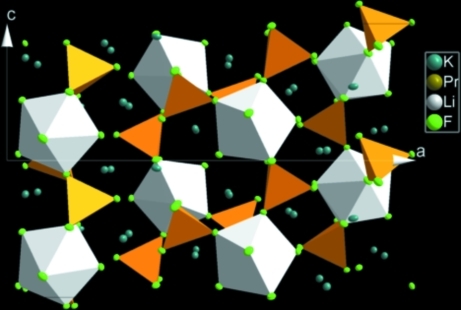
Crystal packing of the K5PrLi2F10 structure as seen down [010]. Displacement ellipsoids have been drawn at the 50% probability level.

## supplementary crystallographic information

### Comment

The title crystal belongs to so-called self-activated materials containing lanthanoid ions within the matrix. An important feature of these systems is a large separation between the closest lanthanoid ions, which is one of the crucial factors governing the self-quenching of luminescence.

In the structure, two different LiF_4_ tetrahedra together with distorted PrF_8_ dodecahedra form sheets expanding perpendicular to [100]. K1 atoms occupy cavities within the sheets and are surrounded by 9 F^-^ ions in a mean distance of 2.780 Å. The remaining potassium atoms are located between the sheets within a KF_9_ and KF_8_ environment. Each PrF_8_ dodecahedron is surrounded by twelve others with a minimum and maximum Pr—Pr separation of 6.7656 (2) and 7.8684 (2) Å, and individual distances of: 2× 6.7656 (2), 2× 6.9169 (2), 2× 6.9255 (2), 2× 7.7903 (3) and 4× 7.8684 (2) Å.

The bond valence sums of all metal atoms have been calculated from the received structure model using the bond-valence method (Brown, 1992, 2002; Mattausch *et al.*, 1991); Pr 2.86, K1 1.05, K2 0.98, K3 1.06, and Li 1.08 v.u. The Pr ion is slightly under-bonded which may be associated with the distorted surrounding of this cation. When such distortions occur, the equal-valence rule is not obeyed (Brown, 1992). The valences of K atoms are close to the formal charge of +1. The Li position is over-bonded with a 8.3% higher bond-valence sum than those expected from the formal charge of +1.

### Experimental

The K_5_PrLi_2_F_10_ crystal was grown from commercially available KF, PrF_3_ and LiF (Aldrich 99.99%, anhydrous) using the Bridgman method in a graphite crucible under argon atmosphere. The reagents were heated at 923 K (melting point 813 K). The pulling rate was 1mm/h, temperature gradient was 100°/cm.

### Refinement

In the final Fourier map, the highest peak is 1.22 Å from atom Pr1 and the deepest hole is 0.55 Å from the same atom.

### Crystal data

**Table:**

K_5_PrLi_2_F_10_	*F*(000) = 1000
*M**_r_* = 540.29	*D*_x_ = 3.221 Mg m^−^^3^
Orthorhombic, *P**n**m**a*	Mo *K*α radiation, λ = 0.71073 Å
Hall symbol: -P 2ac 2n	Cell parameters from 11195 reflections
*a* = 20.6492 (6) Å	θ = 2.6–47.0°
*b* = 7.7903 (3) Å	µ = 6.34 mm^−^^1^
*c* = 6.9255 (2) Å	*T* = 295 K
*V* = 1114.06 (6) Å^3^	Rectangular prism, colourless
*Z* = 4	0.35 × 0.13 × 0.05 mm

### Data collection

**Table:**

Kuma KM-4 with CCD area-detector diffractometer	4910 independent reflections
Radiation source: fine-focus sealed tube	3709 reflections with *I* > 2σ(*I*)
graphite	*R*_int_ = 0.045
Detector resolution: 1024x1024 with blocks 2x2, 33.133pixel/mm pixels mm^-1^	θ_max_ = 47.1°, θ_min_ = 3.1°
ω scans	*h* = −19→42
Absorption correction: multi-scan (*CrysAlis RED*; Oxford Diffraction, 2007)	*k* = −16→13
*T*_min_ = 0.40, *T*_max_ = 0.73	*l* = −13→11
24749 measured reflections	

### Refinement

**Table:**

Refinement on *F*^2^	Primary atom site location: structure-invariant direct methods
Least-squares matrix: full	Secondary atom site location: difference Fourier map
*R*[*F*^2^ > 2σ(*F*^2^)] = 0.029	*w* = 1/[σ^2^(*F*_o_^2^) + (0.0151*P*)^2^] where *P* = (*F*_o_^2^ + 2*F*_c_^2^)/3
*wR*(*F*^2^) = 0.045	(Δ/σ)_max_ = 0.001
*S* = 1.02	Δρ_max_ = 1.17 e Å^−^^3^
4910 reflections	Δρ_min_ = −2.04 e Å^−^^3^
98 parameters	Extinction correction: *SHELXL97* (Sheldrick, 2008), Fc^*^=kFc[1+0.001xFc^2^λ^3^/sin(2θ)]^-1/4^
0 restraints	Extinction coefficient: 0.0319 (3)

### Special details

**Table:**

Geometry. All esds (except the esd in the dihedral angle between two l.s. planes) are estimated using the full covariance matrix. The cell esds are taken into account individually in the estimation of esds in distances, angles and torsion angles; correlations between esds in cell parameters are only used when they are defined by crystal symmetry. An approximate (isotropic) treatment of cell esds is used for estimating esds involving l.s. planes.
Refinement. Refinement of *F*^2^ against ALL reflections. The weighted *R*-factor *wR* and goodness of fit *S* are based on *F*^2^, conventional *R*-factors *R* are based on *F*, with *F* set to zero for negative *F*^2^. The threshold expression of *F*^2^ > σ(*F*^2^) is used only for calculating *R*-factors(gt) *etc*. and is not relevant to the choice of reflections for refinement. *R*-factors based on *F*^2^ are statistically about twice as large as those based on *F*, and *R*- factors based on ALL data will be even larger.To eliminate the weak reflections measured at high theta angles a 2theta limit was applied during structure refinement. The refinement on the whole data set (2theta = 47°) only slightly improved the standard deviations. Concluding, it was decided to refine the structure using a maximum measured 2theta limit. For completeness calculations the 2theta threshold was set to 28.

### Fractional atomic coordinates and isotropic or equivalent isotropic displacement parameters (Å^2^)

**Table:**

	*x*	*y*	*z*	*U*_iso_*/*U*_eq_	
K1	0.456661 (15)	0.97842 (4)	0.25210 (4)	0.01639 (5)	
K2	0.283103 (15)	0.02682 (4)	0.42595 (5)	0.01786 (6)	
K3	0.35981 (2)	0.2500	0.94040 (6)	0.01865 (8)	
Pr1	0.107262 (4)	0.2500	0.239205 (12)	0.00794 (2)	
Li1	0.92241 (16)	0.2500	0.9697 (5)	0.0140 (6)	
Li2	0.67429 (16)	0.2500	0.8399 (5)	0.0138 (6)	
F1	0.00854 (5)	0.2500	0.04643 (17)	0.0152 (2)	
F2	0.01862 (6)	0.2500	0.45774 (17)	0.0189 (2)	
F3	0.09014 (4)	0.95753 (10)	0.15779 (13)	0.01896 (17)	
F4	0.14806 (4)	0.07426 (10)	0.50594 (12)	0.01643 (16)	
F5	0.21956 (6)	0.2500	0.19130 (18)	0.0182 (2)	
F6	0.37398 (6)	0.2500	0.31393 (18)	0.0170 (2)	
F7	0.75970 (6)	0.2500	0.79114 (17)	0.0171 (2)	
F8	0.63138 (6)	0.2500	0.60663 (16)	0.0160 (2)	

### Atomic displacement parameters (Å^2^)

**Table:**

	*U*^11^	*U*^22^	*U*^33^	*U*^12^	*U*^13^	*U*^23^
K1	0.01604 (12)	0.01537 (12)	0.01777 (13)	−0.00135 (9)	−0.00063 (10)	−0.00158 (10)
K2	0.01912 (13)	0.01524 (13)	0.01921 (14)	0.00175 (10)	−0.00062 (11)	−0.00098 (10)
K3	0.0311 (2)	0.01106 (17)	0.01382 (18)	0.000	−0.00294 (15)	0.000
Pr1	0.00890 (4)	0.00760 (4)	0.00733 (4)	0.000	−0.00056 (3)	0.000
Li1	0.0140 (15)	0.0148 (16)	0.0131 (15)	0.000	−0.0021 (12)	0.000
Li2	0.0128 (16)	0.0152 (16)	0.0132 (15)	0.000	0.0005 (12)	0.000
F1	0.0111 (5)	0.0188 (6)	0.0156 (5)	0.000	−0.0023 (4)	0.000
F2	0.0180 (6)	0.0243 (6)	0.0144 (5)	0.000	0.0026 (4)	0.000
F3	0.0249 (4)	0.0109 (4)	0.0211 (4)	0.0000 (3)	−0.0063 (3)	−0.0032 (3)
F4	0.0240 (4)	0.0101 (3)	0.0153 (4)	−0.0001 (3)	−0.0031 (3)	−0.0007 (3)
F5	0.0129 (5)	0.0237 (6)	0.0182 (5)	0.000	−0.0015 (4)	0.000
F6	0.0170 (5)	0.0210 (6)	0.0132 (5)	0.000	−0.0024 (4)	0.000
F7	0.0144 (5)	0.0210 (6)	0.0160 (5)	0.000	0.0017 (4)	0.000
F8	0.0155 (5)	0.0212 (6)	0.0112 (5)	0.000	−0.0015 (4)	0.000

### Geometric parameters (Å, °)

**Table:**

K1—F8^i^	2.7256 (9)	K3—F5^xv^	3.3772 (13)
K1—F1^ii^	2.7514 (8)	Pr1—F5	2.3426 (12)
K1—F2^iii^	2.7537 (10)	Pr1—F3^xviii^	2.3737 (8)
K1—F6^iv^	2.7521 (9)	Pr1—F3^xix^	2.3737 (8)
K1—F4^iii^	2.7841 (9)	Pr1—F2	2.3751 (12)
K1—F1^v^	2.7996 (9)	Pr1—F1	2.4368 (11)
K1—F3^vi^	2.8307 (10)	Pr1—F8^xx^	2.4463 (11)
K1—F2^ii^	2.8679 (9)	Pr1—F4^xxi^	2.4488 (8)
K1—Li1^vii^	2.948 (2)	Pr1—F4	2.4488 (8)
K1—F3^viii^	3.0127 (9)	Pr1—Li2^x^	3.227 (3)
K1—Li2^i^	3.299 (3)	Li1—F6^xvii^	1.802 (4)
K1—Li1^ix^	3.416 (3)	Li1—F1^xxii^	1.856 (4)
K2—F7^x^	2.6636 (9)	Li1—F3^i^	1.8602 (19)
K2—F6	2.6733 (10)	Li1—F3^xxiii^	1.8602 (19)
K2—F5	2.7176 (10)	Li1—K1^xxiv^	2.948 (2)
K2—F7^xi^	2.7735 (8)	Li1—K1^xxv^	2.948 (2)
K2—F8^xi^	2.7964 (8)	Li1—K3^xvii^	3.120 (3)
K2—F5^xii^	2.8338 (9)	Li1—K1^xxvi^	3.416 (3)
K2—F4	2.8669 (9)	Li1—K1^xxvii^	3.416 (3)
K2—Li2^xi^	2.969 (2)	Li1—K2^xvii^	3.438 (3)
K2—F3^v^	3.0730 (10)	Li1—K2^xxviii^	3.438 (3)
K2—Li2^x^	3.271 (3)	Li2—F7	1.796 (4)
K2—F4^xiii^	3.3319 (9)	Li2—F4^xvii^	1.819 (2)
K2—Li1^x^	3.438 (3)	Li2—F4^xxviii^	1.819 (2)
K3—F4^xiv^	2.5717 (8)	Li2—F8	1.843 (4)
K3—F4^xii^	2.5717 (8)	Li2—K2^xi^	2.969 (2)
K3—F6^xv^	2.6035 (13)	Li2—K2^xxix^	2.969 (2)
K3—F7^x^	2.6161 (12)	Li2—Pr1^xvii^	3.227 (3)
K3—F3^v^	2.7409 (9)	Li2—K2^xxviii^	3.271 (3)
K3—F3^xvi^	2.7409 (9)	Li2—K2^xvii^	3.271 (3)
K3—Li1^x^	3.120 (3)	Li2—K1^i^	3.299 (3)
K3—F2^xvii^	3.3544 (13)	Li2—K1^xxiii^	3.299 (3)
			
F8^i^—K1—F1^ii^	125.43 (3)	F4^xxi^—Pr1—F4	67.98 (4)
F8^i^—K1—F2^iii^	88.14 (3)	F6^xvii^—Li1—F1^xxii^	107.07 (18)
F1^ii^—K1—F2^iii^	143.14 (3)	F6^xvii^—Li1—F3^i^	108.50 (12)
F8^i^—K1—F6^iv^	91.85 (3)	F1^xxii^—Li1—F3^i^	105.63 (12)
F1^ii^—K1—F6^iv^	64.63 (3)	F6^xvii^—Li1—F3^xxiii^	108.50 (12)
F2^iii^—K1—F6^iv^	136.63 (3)	F1^xxii^—Li1—F3^xxiii^	105.63 (12)
F8^i^—K1—F4^iii^	66.76 (3)	F3^i^—Li1—F3^xxiii^	120.71 (19)
F1^ii^—K1—F4^iii^	136.58 (3)	F7—Li2—F4^xvii^	113.76 (13)
F2^iii^—K1—F4^iii^	66.13 (3)	F7—Li2—F4^xxviii^	113.76 (13)
F6^iv^—K1—F4^iii^	74.14 (3)	F4^xvii^—Li2—F4^xxviii^	97.67 (17)
F8^i^—K1—F1^v^	59.66 (3)	F7—Li2—F8	107.90 (19)
F1^ii^—K1—F1^v^	91.123 (11)	F4^xvii^—Li2—F8	111.81 (14)
F2^iii^—K1—F1^v^	94.63 (3)	F4^xxviii^—Li2—F8	111.81 (14)
F6^iv^—K1—F1^v^	122.31 (3)	Li1^xxx^—F1—Pr1	163.42 (12)
F4^iii^—K1—F1^v^	123.47 (3)	Li1^xxx^—F1—K1^xxxi^	76.83 (7)
F8^i^—K1—F3^vi^	122.20 (3)	Pr1—F1—K1^xxxi^	92.75 (3)
F1^ii^—K1—F3^vi^	63.47 (3)	Li1^xxx^—F1—K1^xxxii^	76.83 (7)
F2^iii^—K1—F3^vi^	86.88 (3)	Pr1—F1—K1^xxxii^	92.75 (3)
F6^iv^—K1—F3^vi^	127.87 (3)	K1^xxxi^—F1—K1^xxxii^	100.52 (4)
F4^iii^—K1—F3^vi^	151.98 (3)	Li1^xxx^—F1—K1^iii^	92.14 (9)
F1^v^—K1—F3^vi^	63.45 (3)	Pr1—F1—K1^iii^	100.61 (3)
F8^i^—K1—F2^ii^	163.96 (3)	K1^xxxi^—F1—K1^iii^	88.877 (11)
F1^ii^—K1—F2^ii^	61.06 (3)	K1^xxxii^—F1—K1^iii^	163.31 (4)
F2^iii^—K1—F2^ii^	91.081 (12)	Li1^xxx^—F1—K1^xxxiii^	92.14 (9)
F6^iv^—K1—F2^ii^	77.79 (3)	Pr1—F1—K1^xxxiii^	100.61 (3)
F4^iii^—K1—F2^ii^	98.33 (3)	K1^xxxi^—F1—K1^xxxiii^	163.31 (4)
F1^v^—K1—F2^ii^	136.33 (3)	K1^xxxii^—F1—K1^xxxiii^	88.877 (11)
F3^vi^—K1—F2^ii^	73.73 (3)	K1^iii^—F1—K1^xxxiii^	78.93 (3)
F8^i^—K1—F3^viii^	63.86 (3)	Pr1—F2—K1^xvi^	109.20 (4)
F1^ii^—K1—F3^viii^	61.62 (3)	Pr1—F2—K1^v^	109.20 (4)
F2^iii^—K1—F3^viii^	148.97 (3)	K1^xvi^—F2—K1^v^	80.51 (3)
F6^iv^—K1—F3^viii^	61.87 (3)	Pr1—F2—K1^xxxi^	91.20 (3)
F4^iii^—K1—F3^viii^	110.26 (3)	K1^xvi^—F2—K1^xxxi^	159.16 (5)
F1^v^—K1—F3^viii^	60.57 (3)	K1^v^—F2—K1^xxxi^	88.919 (12)
F3^vi^—K1—F3^viii^	96.68 (2)	Pr1—F2—K1^xxxii^	91.20 (3)
F2^ii^—K1—F3^viii^	119.57 (3)	K1^xvi^—F2—K1^xxxii^	88.919 (12)
Li1^vii^—K1—F3^viii^	36.35 (5)	K1^v^—F2—K1^xxxii^	159.16 (5)
F7^x^—K2—F6	85.20 (3)	K1^xxxi^—F2—K1^xxxii^	95.07 (4)
F7^x^—K2—F5	86.26 (3)	Pr1—F2—K3^x^	152.55 (5)
F6—K2—F5	75.48 (3)	K1^xvi^—F2—K3^x^	91.46 (3)
F7^x^—K2—F7^xi^	148.04 (2)	K1^v^—F2—K3^x^	91.46 (3)
F6—K2—F7^xi^	124.90 (3)	K1^xxxi^—F2—K3^x^	70.76 (3)
F5—K2—F7^xi^	91.11 (3)	K1^xxxii^—F2—K3^x^	70.76 (3)
F7^x^—K2—F8^xi^	132.63 (3)	Li1^i^—F3—Pr1^iv^	165.41 (10)
F6—K2—F8^xi^	92.00 (3)	Li1^i^—F3—K3^iii^	83.04 (9)
F5—K2—F8^xi^	138.59 (3)	Pr1^iv^—F3—K3^iii^	109.90 (3)
F7^xi^—K2—F8^xi^	63.76 (3)	Li1^i^—F3—K1^xx^	91.08 (11)
F7^x^—K2—F5^xii^	90.93 (3)	Pr1^iv^—F3—K1^xx^	92.15 (3)
F6—K2—F5^xii^	134.17 (3)	K3^iii^—F3—K1^xx^	104.10 (3)
F5—K2—F5^xii^	149.912 (14)	Li1^i^—F3—K1^xxxiv^	69.92 (9)
F7^xi^—K2—F5^xii^	75.74 (3)	Pr1^iv^—F3—K1^xxxiv^	96.33 (3)
F8^xi^—K2—F5^xii^	58.52 (3)	K3^iii^—F3—K1^xxxiv^	152.20 (3)
F7^x^—K2—F4	66.25 (3)	K1^xx^—F3—K1^xxxiv^	83.32 (2)
F6—K2—F4	130.92 (3)	Li1^i^—F3—K2^iii^	84.56 (11)
F5—K2—F4	64.12 (3)	Pr1^iv^—F3—K2^iii^	87.66 (3)
F7^xi^—K2—F4	83.97 (3)	K3^iii^—F3—K2^iii^	94.32 (3)
F8^xi^—K2—F4	136.82 (3)	K1^xx^—F3—K2^iii^	160.43 (4)
F5^xii^—K2—F4	87.36 (3)	K1^xxxiv^—F3—K2^iii^	77.26 (2)
F7^x^—K2—F3^v^	75.18 (3)	Li2^x^—F4—Pr1	97.15 (9)
F6—K2—F3^v^	61.83 (3)	Li2^x^—F4—K3^xiii^	149.53 (9)
F5—K2—F3^v^	134.29 (3)	Pr1—F4—K3^xiii^	113.22 (3)
F7^xi^—K2—F3^v^	125.84 (3)	Li2^x^—F4—K1^v^	89.01 (11)
F8^xi^—K2—F3^v^	62.28 (3)	Pr1—F4—K1^v^	106.09 (3)
F5^xii^—K2—F3^v^	73.02 (3)	K3^xiii^—F4—K1^v^	85.08 (3)
F4—K2—F3^v^	136.25 (3)	Li2^x^—F4—K2	85.46 (11)
Li2^xi^—K2—F3^v^	95.76 (7)	Pr1—F4—K2	105.13 (3)
F7^x^—K2—F4^xiii^	150.90 (3)	K3^xiii^—F4—K2	84.30 (3)
F6—K2—F4^xiii^	66.48 (3)	K1^v^—F4—K2	148.74 (3)
F5—K2—F4^xiii^	80.51 (3)	Li2^x^—F4—K2^xii^	62.51 (9)
F7^xi^—K2—F4^xiii^	58.60 (3)	Pr1—F4—K2^xii^	159.63 (3)
F8^xi^—K2—F4^xiii^	58.53 (3)	K3^xiii^—F4—K2^xii^	87.07 (3)
F5^xii^—K2—F4^xiii^	113.24 (3)	K1^v^—F4—K2^xii^	76.23 (2)
F4—K2—F4^xiii^	127.88 (3)	K2—F4—K2^xii^	73.936 (19)
Li2^xi^—K2—F4^xiii^	32.91 (5)	Pr1—F5—K2^xxi^	113.16 (4)
F3^v^—K2—F4^xiii^	95.86 (2)	Pr1—F5—K2	113.16 (4)
Li2^x^—K2—F4^xiii^	148.36 (6)	K2^xxi^—F5—K2	79.55 (4)
F4^xiv^—K3—F4^xii^	158.39 (4)	Pr1—F5—K2^xiii^	94.16 (3)
F4^xiv^—K3—F6^xv^	80.31 (2)	K2^xxi^—F5—K2^xiii^	152.13 (5)
F4^xii^—K3—F6^xv^	80.31 (2)	K2—F5—K2^xiii^	84.854 (11)
F4^xiv^—K3—F7^x^	93.34 (2)	Pr1—F5—K2^xxxv^	94.16 (3)
F4^xii^—K3—F7^x^	93.34 (2)	K2^xxi^—F5—K2^xxxv^	84.854 (11)
F6^xv^—K3—F7^x^	134.26 (4)	K2—F5—K2^xxxv^	152.13 (5)
F4^xiv^—K3—F3^v^	136.82 (3)	K2^xiii^—F5—K2^xxxv^	99.10 (4)
F4^xii^—K3—F3^v^	64.56 (3)	Pr1—F5—K3^xxxvi^	157.18 (5)
F6^xv^—K3—F3^v^	131.85 (3)	K2^xxi^—F5—K3^xxxvi^	83.90 (3)
F7^x^—K3—F3^v^	81.97 (3)	K2—F5—K3^xxxvi^	83.90 (3)
F4^xiv^—K3—F3^xvi^	64.56 (3)	K2^xiii^—F5—K3^xxxvi^	71.52 (3)
F4^xii^—K3—F3^xvi^	136.82 (3)	K2^xxxv^—F5—K3^xxxvi^	71.52 (3)
F6^xv^—K3—F3^xvi^	131.85 (3)	Li1^x^—F6—K3^xxxvi^	152.74 (12)
F7^x^—K3—F3^xvi^	81.97 (3)	Li1^x^—F6—K2	98.53 (9)
F3^v^—K3—F3^xvi^	72.29 (4)	K3^xxxvi^—F6—K2	102.09 (4)
F4^xiv^—K3—F2^xvii^	91.41 (2)	Li1^x^—F6—K2^xxi^	98.53 (9)
F4^xii^—K3—F2^xvii^	91.41 (2)	K3^xxxvi^—F6—K2^xxi^	102.09 (4)
F6^xv^—K3—F2^xvii^	71.40 (4)	K2—F6—K2^xxi^	81.14 (4)
F7^x^—K3—F2^xvii^	154.34 (4)	Li1^x^—F6—K1^xix^	77.59 (7)
F3^v^—K3—F2^xvii^	77.38 (3)	K3^xxxvi^—F6—K1^xix^	85.13 (3)
F3^xvi^—K3—F2^xvii^	77.38 (3)	K2—F6—K1^xix^	168.70 (4)
Li1^x^—K3—F2^xvii^	77.66 (7)	K2^xxi^—F6—K1^xix^	88.891 (10)
F4^xiv^—K3—F5^xv^	81.66 (2)	Li1^x^—F6—K1^xviii^	77.59 (7)
F4^xii^—K3—F5^xv^	81.66 (2)	K3^xxxvi^—F6—K1^xviii^	85.13 (3)
F6^xv^—K3—F5^xv^	65.49 (4)	K2—F6—K1^xviii^	88.892 (10)
F7^x^—K3—F5^xv^	68.77 (3)	K2^xxi^—F6—K1^xviii^	168.70 (4)
F3^v^—K3—F5^xv^	133.69 (2)	K1^xix^—F6—K1^xviii^	100.48 (4)
F3^xvi^—K3—F5^xv^	133.69 (2)	Li2—F7—K3^xvii^	153.04 (13)
Li1^x^—K3—F5^xv^	145.44 (7)	Li2—F7—K2^xxviii^	92.28 (9)
F2^xvii^—K3—F5^xv^	136.89 (3)	K3^xvii^—F7—K2^xxviii^	107.91 (4)
F5—Pr1—F3^xviii^	96.53 (2)	Li2—F7—K2^xvii^	92.28 (9)
F5—Pr1—F3^xix^	96.53 (2)	K3^xvii^—F7—K2^xvii^	107.91 (4)
F3^xviii^—Pr1—F3^xix^	147.44 (4)	K2^xxviii^—F7—K2^xvii^	81.49 (4)
F5—Pr1—F2	148.56 (4)	Li2—F7—K2^xi^	77.81 (7)
F3^xviii^—Pr1—F2	92.10 (2)	K3^xvii^—F7—K2^xi^	85.39 (3)
F3^xix^—Pr1—F2	92.10 (2)	K2^xxviii^—F7—K2^xi^	164.59 (4)
F5—Pr1—F1	138.64 (4)	K2^xvii^—F7—K2^xi^	87.078 (7)
F3^xviii^—Pr1—F1	75.24 (2)	Li2—F7—K2^xxix^	77.81 (7)
F3^xix^—Pr1—F1	75.24 (2)	K3^xvii^—F7—K2^xxix^	85.39 (3)
F2—Pr1—F1	72.81 (4)	K2^xxviii^—F7—K2^xxix^	87.078 (7)
F5—Pr1—F8^xx^	70.11 (4)	K2^xvii^—F7—K2^xxix^	164.59 (4)
F3^xviii^—Pr1—F8^xx^	78.33 (2)	K2^xi^—F7—K2^xxix^	102.07 (4)
F3^xix^—Pr1—F8^xx^	78.33 (2)	Li2—F8—Pr1^vi^	163.01 (13)
F2—Pr1—F8^xx^	141.33 (4)	Li2—F8—K1^i^	90.34 (9)
F1—Pr1—F8^xx^	68.53 (4)	Pr1^vi^—F8—K1^i^	102.45 (3)
F5—Pr1—F4^xxi^	76.48 (3)	Li2—F8—K1^xxiii^	90.34 (9)
F3^xviii^—Pr1—F4^xxi^	140.09 (3)	Pr1^vi^—F8—K1^xxiii^	102.45 (3)
F3^xix^—Pr1—F4^xxi^	72.17 (3)	K1^i^—F8—K1^xxiii^	81.52 (3)
F2—Pr1—F4^xxi^	77.55 (3)	Li2—F8—K2^xxix^	76.53 (7)
F1—Pr1—F4^xxi^	134.52 (3)	Pr1^vi^—F8—K2^xxix^	92.84 (3)
F8^xx^—Pr1—F4^xxi^	131.95 (3)	K1^i^—F8—K2^xxix^	162.49 (4)
F5—Pr1—F4	76.48 (3)	K1^xxiii^—F8—K2^xxix^	86.943 (11)
F3^xviii^—Pr1—F4	72.17 (3)	Li2—F8—K2^xi^	76.53 (7)
F3^xix^—Pr1—F4	140.09 (3)	Pr1^vi^—F8—K2^xi^	92.84 (3)
F2—Pr1—F4	77.55 (3)	K1^i^—F8—K2^xi^	86.943 (11)
F1—Pr1—F4	134.52 (3)	K1^xxiii^—F8—K2^xi^	162.49 (4)
F8^xx^—Pr1—F4	131.95 (3)	K2^xxix^—F8—K2^xi^	100.92 (4)

Symmetry codes: (i) −*x*+1, −*y*+1, −*z*+1; (ii) *x*+1/2, *y*+1, −*z*+1/2; (iii) −*x*+1/2, −*y*+1, *z*−1/2; (iv) *x*, *y*+1, *z*; (v) −*x*+1/2, −*y*+1, *z*+1/2; (vi) *x*+1/2, *y*, −*z*+1/2; (vii) *x*−1/2, *y*+1, −*z*+3/2; (viii) −*x*+1/2, −*y*+2, *z*+1/2; (ix) −*x*+3/2, −*y*+1, *z*−1/2; (x) *x*−1/2, *y*, −*z*+3/2; (xi) −*x*+1, −*y*, −*z*+1; (xii) −*x*+1/2, −*y*, *z*+1/2; (xiii) −*x*+1/2, −*y*, *z*−1/2; (xiv) −*x*+1/2, *y*+1/2, *z*+1/2; (xv) *x*, *y*, *z*+1; (xvi) −*x*+1/2, *y*−1/2, *z*+1/2; (xvii) *x*+1/2, *y*, −*z*+3/2; (xviii) *x*, *y*−1, *z*; (xix) *x*, −*y*+3/2, *z*; (xx) *x*−1/2, *y*, −*z*+1/2; (xxi) *x*, −*y*+1/2, *z*; (xxii) *x*+1, *y*, *z*+1; (xxiii) −*x*+1, *y*−1/2, −*z*+1; (xxiv) *x*+1/2, −*y*+3/2, −*z*+3/2; (xxv) *x*+1/2, *y*−1, −*z*+3/2; (xxvi) −*x*+3/2, −*y*+1, *z*+1/2; (xxvii) −*x*+3/2, *y*−1/2, *z*+1/2; (xxviii) *x*+1/2, −*y*+1/2, −*z*+3/2; (xxix) −*x*+1, *y*+1/2, −*z*+1; (xxx) *x*−1, *y*, *z*−1; (xxxi) *x*−1/2, *y*−1, −*z*+1/2; (xxxii) *x*−1/2, −*y*+3/2, −*z*+1/2; (xxxiii) −*x*+1/2, *y*−1/2, *z*−1/2; (xxxiv) −*x*+1/2, −*y*+2, *z*−1/2; (xxxv) −*x*+1/2, *y*+1/2, *z*−1/2; (xxxvi) *x*, *y*, *z*−1.
